# Association between eating behavior and poor glycemic control in Japanese adults

**DOI:** 10.1038/s41598-019-39001-y

**Published:** 2019-03-04

**Authors:** Takahiro Iwasaki, Akiko Hirose, Tetsuji Azuma, Tamie Ohashi, Kazutoshi Watanabe, Akihiro Obora, Fumiko Deguchi, Takao Kojima, Atsunori Isozaki, Takaaki Tomofuji

**Affiliations:** 1 Department of Community Oral Health, School of Dentistry, Asahi University, 1851 Hozumi, Mizuho, Gifu, 501-0296 Japan; 2 Asahi University Hospital, 3- 23 Hashimoto-cho, Gifu, Gifu, 500-8523 Japan; 3 School of Dental Hygienists, Asahi University, 1851 Hozumi, Mizuho, Gifu, 501-0296 Japan

## Abstract

This study investigated the relationship between eating behavior and poor glycemic control in 5,479 Japanese adults with hemoglobin A1c (HbA1c) <6.5% who participated in health checks. Respondents to a 2013 baseline survey of eating behavior, including skipping breakfast and how quickly they consumed food were followed up until 2017. We defined poor glycemic control after follow-up as HbA1c ≥6.5%, or increases in HbA1c of ≥0.5% and/or being under medication to control diabetes. We identified 109 (2.0%) respondents who met these criteria for poor glycemic control. After adjusting for sex, age, smoking status, body mass index (BMI), and eating behavior, the risk of poor glycemic control was increased in males (odds ratio [OR], 2.38; 95% confidence interval [CI] 1.37–4.12; *p* < 0.01), and associated with being older (OR, 1.07; 95% CI, 1.04–1.11; *p* < 0.001), having a higher BMI (OR, 1.29; 95% CI 1.23–1.35; *p* < 0.001), skipping breakfast ≥3 times/week (OR, 2.44; 95% CI, 1.35–4.41; *p* < 0.01), and changing from eating slowly or at medium speed to eating quickly (OR, 2.11; 95% CI, 1.04–4.26; *p* < 0.05). In conclusion, Japanese adults who were male, older, had a high BMI, skipped breakfast ≥3 times/week and ate quickly were at increased risk for poor glycemic control.

## Introduction

Diabetes mellitus is characterized by chronic hyperglycemia and is closely associated with many serious health issues, including neuropathy, nephropathy, and retinopathy^1^. The global prevalence of type 2 diabetes has increased from 8.1% to 9.6% in Asians and from 6.0% to 7.9% in Caucasians^2^. The reported incidence of type 2 diabetes in Japan increased from 6.9 to 10 per million between 1997 and 2016^3^. Because diabetes and its associated complications comprise a major health concern, the Japanese government has continuously strived to establish a national policy to prevent diabetes.

Hemoglobin A1c (HbA1c) reflects glycemic status over time and thus serves as a biomarker for testing and monitoring diabetes^4^. It can be applied to guide strategies for treating and controlling diabetes and to predict the risk of progressive complications of diabetes^5^. Therefore, monitoring HbA1c is important for developing appropriate strategies to prevent diabetes and its associated complications.

Eating behavior, such as skipping breakfast and rapidly consuming food, are lifestyle factors that could contribute to an increased risk of diabetes. For instance, a meta-analysis found a pooled adjusted relative risk between skipping breakfast and type 2 diabetes of 1.21 (95% CI, 1.05–1.24)^6^. In addition, a case control study associated a higher risk of diabetes with rapid, rather than slow food consumption^7^. A cross-sectional study also associated hyperglycemia in the general population with late-night food consumption^8^. These findings indicated that eating behavior is associated with risk of diabetes. However, clinical studies are required because evidence of risk for poor glycemic control is limited.

The Japanese Ministry of Health, Labor and Welfare has recommended specific health checks that focus on metabolic syndrome for individuals aged 40–74 years. Medical insurers systematically implement regular health checks, survey eating behavior and measure HbAlc levels. Therefore, the research on Japanese adults was convenient to collect information on eating behavior and HbA1c level. The present study tested the hypothesis that eating behavior is associated with a significantly increased risk of poor glycemic control in a Japanese population who underwent regular health checks.

## Results

The baseline prevalence of skipping breakfast ≥3 times/week and rapid food consumption were 9.7% and 41.5%, respectively (Table 1). Study participants who skipped breakfast ≥3 times/week were significantly more likely to be male, older, exercise less frequently, have a higher body mass index (BMI), higher HbA1c values, a higher frequency of dinner within two hours of bedtime, a higher frequency of eating snacks after dinner and consume more alcohol, than those who did not (*p* < 0.05 for all). Compared with participants that did not eat quickly, those who did were significantly more likely to be male, have higher Brinkman indexes, higher BMI, higher HbA1c, slower walking speed, and higher frequencies of consuming dinner within two hours of bedtime, and of eating snacks after dinner (*p* < 0.05 for all).

**Table 1:** Baseline characteristics of study participants.

Variables	All (n = 5479)	Skipping breakfast		Eating speed	
		<3 times/week (n = 4950)	≥3 times/week (n = 529)	Slowly or medium (n = 3203)	fast (n = 2276)
Male (%)	3380 (61.7)	3003 (60.7)	377 (71.3)^*^	1844 (57.6)	1536 (67.5)^†^
Age (years)	49 (45, 54)	49 (45, 54)	48 (44, 53)^*^	49 (44, 54)	49 (45, 53)
Brinkman index	0 (0, 300)	0 (0, 280)	200 (0, 500)^*^	0 (0, 290)	0 (0, 340)^†^
BMI (kg/m^2^)	22.4 (20.4, 24.6)	22.3 (20.4, 24.5)	22.9 (20.9, 25.3)^*^	21.8 (20.0, 23.8)	23.2 (21.3, 25.6)^†^
HbA1c (%)	5.4 (5.2, 5.5)	5.4 (5.2, 5.5)	5.3 (5.2, 5.5)^*^	5.3 (5.2, 5.5)	5.4 (5.2, 5.6)^†^
Regular exercise habits^b^	1054 (19.2)	977 (19.7)	77 (14.6)^*^	593 (18.5)	461 (20.3)
Walking speed^c^	2659 (48.5)	2402 (48.5)	257 (48.6)	1776 (55.4)	883 (38.8)^†^
Drinking habits^d^	1417 (25.9)	1249 (25.2)	168 (31.8)^*^	821 (25.6)	596 (26.2)
Dinner within 2 hours of bedtime ≥3 times/week^b^	1800 (32.9)	1519 (30.7)	281 (53.1)^*^	915 (28.6)	885 (38.9)^†^
Eating snacks after dinner ≥3 times/week^b^	1049 (19.1)	923 (18.6)	126 (23.8)^*^	531 (16.6)	518 (22.8)^†^

Continuous variables are expressed as median (first quartile, third quartile).

^a^Male (percentage of male); ^b^yes (percentage of yes); ^c^slowly (percentage of slowly); ^d^every day (percentage of every day).

^*^p < 0.05, compared with the participants who skip breakfast <3 times/day, using the chi-square test or the Mann-Whitney *U* test.

^†^p < 0.05, compared with the participants who eat slowly or medium, using the chi-square test or the Mann-Whitney U test.

Abbreviations: BMI, body mass index; HbA1c, hemoglobin A1c.

Ninety (1.6%) of the participants had HbA1c ≥6.5% and 173 (31.6%) had increases in HbA1c of ≥0.5% (Table 2). Among the latter, 46.2% had HbA1c ≥6.5%. The number of participants with diabetes under control with medication was 47 (0.9%). Thus, 109 (2.0%) participants had poor glycemic control at the time of follow up.

**Table 2:** Number of participants with <0.5% or ≥0.5% increase in HbA1c and HbA1c ≥6.5% after followup.

		HbA1c	
		<6.5%	≥6.5%
**Total**			
Increase of HbA1c	<0.5%	5296	10
	0.5%≥	93	80
**With medicine of diabetes**			
Increase of HbA1c	<0.5%	23	2
	≥0.5%	4	18
**Without medicine of diabetes**			
Increase of HbA1c	<0.5%	5273	8
	≥0.5%	89	62

The results of univariate logistic regression analyses showed that odds ratios (OR) for having poor glycemic control were higher for males, older persons, and those with a higher Brinkman index than participants who did not have these factors (*p* < 0.001 for all) (Table 3). ORs for having poor glycemic control were higher when breakfast was skipped ≥3, compared with <3 times/week between baseline and at four years later (OR, 2.57; *p* < 0.01). ORs for having poor glycemic control were increased among those who did not eat quickly at baseline but ate quickly four years later (OR, 2.36; *p* < 0.05) and who ate quickly at baseline and after four years (OR, 2.46; *p* < 0.001). ORs for having poor glycemic control were also higher for consuming snacks after dinner ≥3 times/week at baseline, and eating snacks <3 times/week after four years compared with eating snacks after dinner <3 times/week at baseline and after four years (OR, 1.79; *p* < 0.05).

**Table 3:** Crude odds ratios and 95% CI for poor glycemic control according to factors at baseline and four years later.

Variables		Crude ORs	95% CI	*p* value
Sex	Female	1	(reference)	
	Male	3.68	2.16–6.28	<0.001
Age		1.06	1.03–1.09	<0.001
Brinkman Index		1.001	1.001–1.002	<0.001
Regular exercise habits	No (baseline) & No (after 4 years)	1	(reference)	
	Yes (baseline) & No (after 4 years)	0.77	0.31–1.92	0.57
	No (baseline) & Yes (after 4 years)	0.79	0.38–1.64	0.79
	Yes (baseline) & Yes (after 4 years)	1.54	0.94–2.51	0.09
Walking speed	Fast (baseline) & Fast (after 4 years)	1	(reference)	
	Slowly (baseline) & Fast (after 4 years)	0.58	0.25–1.37	0.21
	Fast (baseline) & Slowly (after 4 years)	1.18	0.61–2.29	0.63
	Slowly (baseline) & Slowly (after 4 years)	1.05	0.69–1.59	0.82
Drinking habits	Not everyday (baseline) & Not everyday (after 4 years)	1	(reference)	
	Everyday (baseline) & Not everyday (after 4 years)	0.95	0.38–2.37	0.91
	Not everyday (baseline) & Everyday (after 4 years)	0.97	0.39–2.42	0.95
	Everyday (baseline) & Everyday (after 4 years)	0.72	0.43–1.21	0.21
Skipping breakfast	<3 times/week (baseline) & <3 times/week (after 4 years)	1	(reference)	
	≥3 times/week (baseline) & <3 times/week (after 4 years)	1.08	0.39–2.97	0.88
	<3 times/week (baseline) & ≥3 times/week (after 4 years)	0.65	0.16–2.67	0.55
	≥3 times/week (baseline) & ≥3 times/week (after 4 years)	2.57	1.47–4.49	<0.01
Eating speed	Slowly or medium (baseline) & Slowly or medium (after 4 years)	1	(reference)	
	Fast (baseline) & Slowly or medium (after 4 years)	1.38	0.61–3.13	0.44
	Slowly or medium (baseline) & Fast (after 4 years)	2.36	1.19–4.69	<0.05
	Fast (2013) & Fast (after 4 years)	2.46	1.61–3.77	<0.001
Dinner within 2 hours of bedtime	<3 times/week (baseline) & <3 times/week (after 4 years)	1	(reference)	
	≥3 times/week (baseline) & <3 times/week (after 4 years)	0.94	0.47–1.89	0.86
	<3 times/week (baseline) & ≥3 times/week (after 4 years)	1.01	0.52–1.97	0.97
	≥3 times/week (baseline) & ≥3 times/week (after 4 years)	1.19	0.66–2.15	0.57
Eating snacks after dinner	<3 times/week (baseline) & <3 times/week (after 4 years)	1	(reference)	
	≥3 times/week (baseline) & <3 times/week (after 4 years)	1.79	1.07–3.01	<0.05
	<3 times/week (baseline) & ≥3 times/week (after 4 years)	0.78	0.35–1.72	0.53
	≥3 times/week (baseline) & ≥3 times/week (after 4 years)	1.28	0.80–2.04	0.31

Abbreviations: ORs, odds ratios; CI, confidence interval.

The risk of having poor glycemic control was increased in males (OR, 2.38; p < 0.01), older persons (OR, 1.07; *p* < 0.001) and those who skipped breakfast ≥3 times/week at baseline and after four years vs. <3 times/week at baseline and after four years vs. (OR, 2.44; *p* < 0.01), those who did not eat quickly at baseline and after four years vs. those who did not eat quickly at baseline but did so after four years (OR, 2.42, *p* < 0.05) and those who did not eat quickly at baseline but did so after four years vs. those who ate quickly at baseline and after four years (OR, 2.31, *p* < 0.001) after adjusting for sex, age, Brinkman index, eating fast, skipping breakfast and eating snacks after dinner (Table 4).

**Table 4:** Adjusted odds ratios and 95% CI for poor glycemic control according to factors at baseline and four years later.

Variables		Adjusted ORs	95% CI	*p* value
**Multivariable-adjusted model** ^a^				
Sex	Female	1	(reference)	
	Male	3.08	1.80–5.28	<0.001
Age		1.06	1.03–1.09	<0.001
Skipping breakfast	<3times/week (baseline) & <3times/week (after 4 years)	1	(reference)	
	≥3times/week (baseline) & <3times/week (after 4 years)	1.04	0.37–2.87	0.95
	<3times/week (baseline) & ≥3times/week (after 4 years)	0.69	0.17–2.87	0.61
	≥3times/week (baseline) & ≥3times/week (after 4 years)	2.44	1.38–4.33	<0.01
Eating speed	Slowly or medium (baseline) & Slowly or medium (after 4 years)	1	(reference)	
	Fast (baseline) & Slowly or medium (after 4 years)	1.22	0.54–2.78	0.64
	Slowly or medium (baseline) & Fast (after 4 years)	2.42	1.21–4.84	<0.05
	Fast (baseline) & Fast (after 4 years)	2.31	1.50–3.55	<0.001

^a^Adjusting sex, age, Brinkman index, skipping breakfast, eating speed, eating snacks after dinner.

Abbreviations: ORs, odds ratios; CI, confidence interval.

## Discussion

This study assessed the relationship between eating behavior and poor glycemic control among Japanese adults who presented for routine health checks. The results indicated that a high frequency of skipping breakfast as well as being male, older and rapidly consuming food predicted risk of poor glycemic control in the present population. These findings were consistent with previous cross-sectional^9,10^ and cohort^11–13^ studies that associated skipping breakfast with a possible risk of diabetes.

Accumulating evidence has associated skipping breakfast with the possibility of poor glycemic control in patients with type 2 diabetes. For instance, skipping breakfast is significantly associated with higher HbA1c values, even after adjusting for age, sex, race, BMI, number of diabetes complications, insulin therapy, depressive symptoms, perceived sleep debt, and ratios (%) of daily caloric intake at dinner in patients with type 2 diabetes^14^. Habitually skipping breakfast concomitant with late evening meals might affect the ability of young male workers with type 2 diabetes to achieve and maintain glycemic control^15^. Furthermore, a randomized clinical trial found that skipping breakfast increased postprandial hyperglycemia after lunch and dinner in patients with diabetes^16^. On the other hand, the present study identified a positive relationship between a high frequency of skipping breakfast and poor glycemic control. The previous and present findings suggest that eating breakfast is important to maintain optimal glycemic control.

Omitting breakfast might reduce free-living physical activity and endurance exercise performance throughout the day^17–19^. Skipping breakfast could increase the risk of poor glycemic control through decreasing in physical activity and performance. In addition, a randomized controlled crossover trial found more 24-h energy expenditure on days when breakfast was skipped (+41 kcal/day) compared with control days when three meals were consumed^20^.

Our findings also showed that the risk of poor glycemic control was increased in participants who ate quickly after adjustment for sex, age, Brinkman index, regular exercise, skipping breakfast and having dinner within two hours of bedtime. Changing from not eating quickly to rapidly eating meals also persisted as a risk factor for poor glycemic control. Previous studies have positively correlated eating quickly with increased BMI^21,22^, which is an established risk factor for the progression of diabetes-associated complications^23,24^. Eating quickly might indirectly increase risk for poor glycemic control through increasing BMI.

Furthermore, we found here that risk of poor glycemic control did not significantly correlate with the frequency of consuming dinner within two hours of bedtime and eating snacks after dinner except when eating snacks after dinner changed from ≥3 to <3 times/week (Table 3). These findings differ from previous results in which the OR of late-night dinner consumption and hyperglycemia defined as HbA1c ≥5.7% and/or pharmacotherapy for diabetes was 1.12^8^. Our definition of poor glycemic control differed from theirs. The relationship between late food consumption and glycemic status might vary according to the definition of poor glycemic control. In addition, our survey of the frequency of consuming dinner within two hours of bedtime and eating snacks after dinner might have a social desirability bias, suggesting that differences between the previous and present observations might be due to methodological issues associated. More data about meal patterns from a food diary, are needed to resolve this issue.

We excluded 481 (5.3%) patients with poor glycemic control at baseline and the prevalence after followup was about 2.0%. A previous cohort study found that the age-standardized prevalence of diabetes among Japanese adults aged 55–74 years was initially 8.2%, and 10.6% five years later^25^. Another study of Japanese adult men and women aged 20–69 years also found 8.0% and 3.3% prevalences of diabetes, respectively^26^. These findings collectively suggest that the prevalence of poor glycemic control in the present study population was relatively low. We recruited participants from a population who attended health checks at Asahi University Hospital. Since this cohort might have been particularly aware of or concerned with health, the ability to extrapolate our findings to the general population would be limited.

This study has other limitations. The validity of the questionnaire regarding eating behavior other than eating speed was not assessed. The data were limited because the data were derived from health-check findings. In addition, data regarding the quality of dietary intake and the frequency of consuming confectionery or sweet snacks will be required to improve the validity of our results. Furthermore, the participants defined dinner, snacks and breakfast. Therefore, the rationale for the ≥3 times/week cut-off for dinner within two hours of bedtime, eating snacks after dinner, and skipping breakfast will require confirmation.

In conclusion, the present findings of this study indicated positive associations between poor glycemic control and skipping breakfast ≥3 times/week, as well as between eating quickly, after adjusting for sex, age, Brinkman index and eating behavior.

## Methods

We analyzed data from community residents who participated in health checks at Asahi University Hospital in Gifu, Japan. A total of 9,132 Japanese adults aged ≥40 years participated in the baseline survey between January and December 2013. We excluded 530 residents without HbA1c data (n = 49), with HbA1c ≥6.5% (n = 324), and those under medication for diabetes and/or insulin therapy (n = 157). We excluded 48 residents because of missing information about eating behavior. Among 8,554 residents, 5,479 were followed up between January and December 2017 (follow-up rate, 64%). Accordingly, we analyzed data from 5,479 community residents in this study, which was approved by the Ethics Committee at Asahi University (No. 27010) and proceeded in accordance with the Declaration of Helsinki. All the residents provided written informed consent to participate in the study.

### Measurement of HbA1c

We determined HbA1c values using the diabetes automatic analyzer (DM-JACK, Kyowa Medex, Tokyo, Japan) in venous blood samples collected after an overnight fast^27,28^.

### Evaluation of poor glycemic control

In general, 0.5% HbA1c is considered a clinically significant change^29^ and ≥6.5% indicates poor glycemic control^30^. Therefore, our participants with HbA1c that reached ≥6.5% with increases of ≥0.5% were defined as having poor glycemic control at follow-up. In addition, some participants received diabetes medications after follow-up, although they did not meet our definition of poor glycemic control. However, since the participants who were under medication had already been diagnosed with diabetes, they were also included in the group with poor glycemic control.

### Assessment of body composition

Height and body weight were measured using an automatic height scale with body composition meters (TBF-110/TBF-210/DC-250; Tanita Corp., Tokyo, Japan)^27^. We calculated BMI as weight (kg) divided by height squared (m^2^).

### Questionnaire

We used the same questionnaire as is used for the health check-ups in Japan. Information about age, sex, presence or absence of regular exercise (absence/presence), walking speed (slow/not slow), alcohol consumption (every day/not every day), eating behavior comprising speed (slowly, medium, or quickly), dinner within two hours of bedtime ≥3 times/week (yes/no), eating snacks after dinner ≥3 times/week (yes/no), and skipping breakfast ≥3 times/week (yes/no)^31,32^. We previously validated self-reported quick eating in the questionnaire^33^. In that study, participants who reported quickly spent less time chewing (rice balls) than those who reported eating slowly or a medium speed).

### Evaluation of smoking status

The Brinkman index (daily number of cigarettes × years) was recorded as their smoking status^34^.

### Statistical analysis

The normality of our data was confirmed using Kolmogorov-Smirnov tests. Because all continuous variables were not normally distributed, data are expressed as medians (first and third quartiles). Significant differences in selected characteristics between study participants who skipped breakfast ≥3 times/week or not and ate rapidly were assessed at baseline using chi-square tests and Mann-Whitney *U* tests. Univariate and multivariate stepwise logistic regression analyses proceeded with the presence or absence of poor glycemic control as dependent variables. Variables with *p* < 0.10 were removed from the model and those with *p* < 0.05 were added. The model included groups with different types of behavior as the independent variable (Table 3). Other independent variables with *p* < 0.05 in the univariate model were selected. In addition, a variable with |r| > 0.8 in the spearman correlation analysis of between each variable was removed to avoid multicollinearity^35^. The ORs and their 95% confidence intervals (95% CI) were calculated for each potential risk factor. All data were analyzed using SPSS statistics version 24 (IBM Japan, Tokyo, Japan). All reported values with *p* < 0.05 were considered statistically significant.
